# Elevated Lipoprotein-Associated Phospholipase A2 Is Associated With Intracranial Atherosclerosis

**DOI:** 10.3389/fneur.2022.858302

**Published:** 2022-06-10

**Authors:** Yuan Wang, Gang Liu, Haiqing Song, Catherine Cao, Xunming Ji, Guodong Cao

**Affiliations:** ^1^Department of Neurology, Xuanwu Hospital, Capital Medicine University, Beijing, China; ^2^Department of Neurology, University of Pittsburgh, Pittsburgh, PA, United States; ^3^Department of Neurosurgery, Xuanwu Hospital, Capital Medicine University, Beijing, China; ^4^Geriatric Research Education and Clinical Center, VA Pittsburgh Healthcare System, Pittsburgh, PA, United States

**Keywords:** acute ischemic stroke, intracranial atherosclerosis, homocysteine, lipoprotein-associated phospholipase A2, stenosis

## Abstract

**Background:**

Lipoprotein-associated phospholipase A2 (Lp-PLA2) is an inflammatory factor in the pathogenesis of atherosclerotic plaque and is associated with an increased risk of ischemic stroke. Whether Lp-PLA2 is associated with stenosis subtypes in acute ischemic stroke (AIS) has not been investigated.

**Methods:**

A total of 126 eligible AIS patients were divided into four groups: (1) no cerebral artery stenosis (NCS); (2) intracranial artery stenosis (ICAS); (3) extracranial artery stenosis (ECAS); and (4) combined intracranial and extracranial artery stenosis (IECS). Associations between serum Lp-PLA2 levels and the stenosis subtypes were assessed.

**Results:**

The ICAS group had a lower frequency of dyslipidemia as compared to the NCS group and the IECS group (35.3% vs. 70% vs. 71.8%, respectively, *p* = 0.001) and was more likely to be symptomatic than the ECAS group (76.5% vs. 43.8%, respectively, *p* = 0.014). Lp-PLA2 levels in the ICAS group were 112.2 ± 66.8 μg/L which are, higher than those in the NCS, ECAS, and IECS groups (81.7 ± 38.5, 106.1 ± 57.8, 89.3 ± 52.2 μg/L, respectively, *p* = 0.025). In the third and fourth quartiles of Lp-PLA2 levels, stenosis had occurred more frequently in the ICAS group than in the other three groups (third Q: 50.0% vs. 3.1% vs. 28.1% vs. 18.8%, *p* = 0.002; fourth Q: 48.4% vs. 16.1% vs. 25.8% vs. 9.7%, *p* = 0.014). Lp-PLA2 levels were higher in patients with more or severe stenosis in the ICAS group.

**Conclusions:**

Elevated Lp-PLA2 levels were differentially associated with increased risk in AIS patients with ICAS compared to those with ECAS or no stenosis. Lp-PLA2 may be a promising biomarker and potential therapeutic target for ICAS.

## Introduction

Stroke is one of the major causes of disability and mortality worldwide. Intracranial atherosclerotic disease (ICAD) has a higher prevalence in the Chinese than in other populations, accounting for approximately one-third of stroke cases in China (1). Patients with ICAD are at a higher risk of recurrent ischemic events and death compared to patients with other stroke subtypes (2).

Lipoprotein-associated phospholipase A2 (Lp-PLA2) plays a key role in the pathogenesis of atherosclerosis due to its pro-inflammatory and pro-oxidative effects. Because of its highly sensitive and specific features for vascular inflammation, Lp-PLA2 raises many concerns and has been linked to an increased risk of first-ever or recurrent ischemic stroke and myocardial infarction (3). As such, the US Food and Drug Administration approved the Lp-PLA2 activity test to evaluate the risk of cardiovascular events (4). The American Heart Association/American Stroke Association demonstrated that Lp-PLA2 measurements are useful in predicting which patients are at higher risk of stroke (5), and the European Society of Cardiology recommended Lp-PLA2 as a predictor of increased risk for a recurrent acute atherothrombotic event (6). A meta-analysis of 32 prospective studies indicated a positive association between Lp-PLA2 activity or levels and the incidences of coronary heart disease, stroke, and cardiovascular disease mortality, after adjustment for traditional risk factors (7). In a stroke-free hypertension cohort, Wang et al. (8) found that Lp-PLA2 levels are significantly related to isolated intracranial artery stenosis (ICAS) and concurrent extra-intracranial stenosis, but not to isolated extracranial artery stenosis (ECAS). In another stroke-free cohort (9), Lp-PLA2 levels were found to be positively correlated with ICAS or ECAS. However, it is unknown whether Lp-PLA2 levels are associated with ICAS in patients with acute ischemic stroke (AIS). Here, we identified an association between serum Lp-PLA2 levels and subtypes of large-vessel atherosclerosis in patients with ischemic stroke.

## Materials and Methods

### Subjects

In this cross-sectional study, we retrospectively reviewed the prospectively maintained stroke registry at Xuanwu Hospital Capital Medical University in China. We prospectively enrolled patients with AIS between March 2010 and February 2012 and included 126 consecutive patients whose Lp-PLA2 levels were obtained within the first 14 days after AIS. Ischemic stroke was diagnosed based on the AHA/ASA criteria (10), and each stroke case was confirmed by brain computed tomography (CT) or magnetic resonance imaging (MRI). The inclusion criteria were as follows: (1) patients with AIS within 14 days from the onset of symptoms and (2) patients aged between 40 and 80 years old. Prior studies (11, 12) showed that thrombolytic therapy and endovascular interventions could alter immune-inflammatory responses after acute stroke. To exclude the impact of revascularization procedures on serum biomarkers, patients were excluded if they had been treated with revascularization procedures within 24 h prior to enrollment. The other exclusion criteria were as follows: (1) any intracranial hemorrhage occurring within 90 days prior to enrollment; (2) arterial stenosis or occlusion due to arterial dissection, due to any known vasculitic disease or hereditary vessel disorders; (3) intracranial neoplasm, cerebral aneurysm, or arteriovenous malformation; (4) cardiac sources of embolism; (5) significant bleeding/coagulation dysfunction; (7) severe renal or hepatic disease, cancer, or critical illness; (7) respiratory and circulatory failure; and (8) acute or chronic inflammatory diseases, and autoimmune diseases. The classification of stroke etiology was based on the Trial of Org 10172 in Acute Stroke Treatment (TOAST) criteria (13). Patients classified with large-artery atherosclerosis (LAA) or small-vessel occlusion (SVO) were included, and the other three subtypes (cardioembolism, stroke of other determined etiology, and stroke of undetermined etiology) were excluded. This study was approved by the Ethics Committees of Xuanwu Hospital, Capital Medical University. All participants signed informed consent forms before participating in this study.

### Clinical Assessments

Clinical information and cerebrovascular risk factors were collected by an interviewer, including personal and family history of hypertension, hyperlipidemia, diabetes and coronary heart disease, smoking and drinking habits, current drug intake, among others. Each patient's initial National Institute of Health Stroke Scale (NIHSS) was assessed at admission. All biochemical measurements, including complete blood count, serum lipid levels, liver and kidney functions, fasting plasma glucose, homocysteine (HCY), and erythrocyte sedimentation rate (ESR) were performed at the Central Laboratory of Xuanwu Hospital (Beijing, China) using standard protocols.

Blood samples were collected within 24 h of admission. Serum was separated by centrifugation for 10 min at 2,000 × g and stored in freezers at −80°C. To reduce inter-assay errors, Lp-PLA2 and high-sensitivity C-reactive protein (hs-CRP) levels were measured simultaneously for all participants using an enzyme-linked immunoassay according to the manufacturer's instructions. In brief, the serum was added to a microtiter plate that was pre-immobilized with a monoclonal antibody against Lp-PLA2 or hs-CRP (DPLG70, QK1707, R&D System, USA) and then incubated with anti-Lp-PLA2 or anti-hs-CRP antibody labeled with horseradish peroxidase. Optical density was determined with a microplate reader at 450 nm.

### CT Angiography Protocol

Computed tomography angiography (CTA) was performed with a 64-section helical CT scanner (GE, Light Speed VCT-XT) as described in an earlier study (14) with modifications. In brief, CTA acquisitions were obtained after a single bolus intravenous injection of 60 ml of iopromide. Scanning covered the whole brain down to the level of the aortic arch with a slice thickness of 5 mm. All images were read with the AW4.2P vessel software independently by two experienced radiologists who were blinded to the clinical data.

The percentage of stenosis was calculated as the ratio of the diameter of the diseased artery at its most severe site divided by the diameter of a nearby normal segment, as previously reported (15). The degree of stenosis was categorized into grades I (30–49%), II (50–69%), and III (≥70%). The number of arteries with stenosis for each patient was counted. The maximum extent of stenosis was chosen as representative of each subject. Based on CTA results, all participants were categorized into four groups (15, 16): no cerebral artery stenosis (NCS), ICAS, ECAS, and combined intracranial and extracranial artery stenosis (IECS). According to the TOAST classification (13), patients with AIS in the NCS group were considered as the TOAST subtype of SVO. Patients with symptomatic artery stenosis in the ICAS, ECAS, or the IECS groups were identified as the subtype of LAA, and the remaining individuals were classified as the subtype of SVO.

### Statistical Analyses

Statistical analyses were performed with SPSS, version 22.0 (SPSS, Inc., Chicago, IL, USA). Data normality was assessed using the Shapiro–Wilk or Kolmogorov–Smirnov test. Continuous variables are presented as mean ± standard deviation (SD) or as medians [interquartile range (IQR)], based on whether the data were normally distributed or not. One-way analysis of variance (ANOVA) test or analysis of covariance for normally distributed continuous variables and the least significant difference (LSD) test for *post-hoc* multiple comparisons were performed. Friedman's test was used in cases where the data were not normally distributed. Categorical variables were compared using chi-squared tests. A Pearson χ^2^ test was used when no subgroup had an expected count below five; otherwise, a Fisher's exact test was performed. The values of *p* < 0.05 were considered statistically significant.

## Results

### Characteristics of the Subject Population

A total of 126 patients with AIS were enrolled in this study. The average age was 59.1 ± 10.2 years, and 78.6% (99 out of 126) were men. Among 126 patients with AIS, 15.9% (*n* = 23) had no stenosis, 40.5% (*n* = 51) had ICAS only, 12.7% (*n* = 16) had ECAS only, and 31.0% (*n* = 39) had IECS. Among the 106 patients with stenosis, 271 arteries were confirmed to be stenotic (Table 1). There were no statistical differences in the proportion of different stenosis grades among the ICAS, ECAS, and IECS groups (*p* = 0.961).

**Table 1:** Numbers, percentages, and grades of arteries with stenosis in the IECS, ICAS, and ECAS groups.

	**IECS group (*****n*** **=** **39)**		**ICAS group** **(*n* = 51)**	**ECAS group** **(*n* = 16)**
	**Intracranial artery**	**Extracranial artery**		
Grade 1 (30%-49%)	29 (30.5%)	21 (32.4%)	25 (28.4%)	8 (34.8%)
Grade 2 (50%-69%)	28 (29.5%)	18 (27.6%)	28 (31.8%)	7 (30.4%)
Grade 3 (≥70%)	38 (40%)	26 (40%)	35 (39.8%)	8 (34.8%)

*The number in front of parentheses is the number of arteries with stenosis*.

The general characteristics of patients according to the location of stenosis are shown in Table 2. Compared to the NCS group, the IECS group had a higher frequency of coronary heart disease (35.9% vs. 5.0%, *p* = 0.01) and a lower frequency of hyperhomocysteinemia (17.9% vs. 50%, *p* = 0.01). The NCS and IECS groups were more likely to suffer from dyslipidemia than the ICAS group (70.0% vs. 71.8% vs. 35.3%, *p* = 0.001). The ICAS group was more likely to be symptomatic compared to the ECAS group (76.5% vs. 43.8%, *p* = 0.014).

**Table 2:** Clinical characteristics of patients according to the location of arterial stenosis.

**Baseline characteristics**	**NCS (*n* = 20)**	**IECS (*n* = 39)**	**ICAS (*n* = 51)**	**ECAS (*n* = 16)**	**x^**2**^/F**	***P***
Age (years)	57.4 ± 12.8	59.9 ± 9.0	57.7 ± 10.3	63.3 ± 8.0	1.531	0.210
Gender (male/female)	15/5	31/8	39/12	14/2	1.062	0.786
NIHSS	4.6 ± 2.7	6.2 ± 4.0	5.2 ± 4.2	4.3 ± 3.3	1.334	0.267
Duration from symptom onset to blood collection (days)	6.5 ± 3.0	6.6 ± 3.5	5.6 ± 3.1	5.9 ± 2.5	0.879	0.454
Hypertension, *n* (%)	15 (75%)	28 (71.8%)	38 (74.5%)	12 (75%)	0.121	0.989
Dyslipidemia, *n* (%)	14 (70%)	28 (71.8%)	18 (35.3%)	6 (37.5%)	15.722	0.001
Diabetes mellitus, *n* (%)	6 (30%)	22 (56.4%)	19 (37.3%)	5 (31.3%)	5.738	0.125
Coronary heart disease, *n* (%)	1 (5%)	14 (35.9%)	8 (15.7%)	2 (12.5%)	10.187	0.017
Hyperhomocysteinemia, *n* (%)	10 (50%)	7 (17.9%)	14 (27.5%)	7 (43.8%)	8.079	0.044
Smoking, *n* (%)	12 (60%)	23 (59%)	26 (51%)	13 (81.3%)	4.626	0.201
Drinking, *n* (%)	11 (55%)	14 (35.9%)	21 (41.2%)	6 (37.5%)	2.114	0.549
Symptomatic artery stenosis, *n* (%)	0	27 (69.2%)	39 (76.5%)	7 (43.8%)	38.099	<0.001
TOAST classification					38.099	<0.001
Large arterial atherosclerosis	0	27 (69.2%)	39 (76.5%)	7 (43.8%)		
Small-vessel occlusion	20 (100%)	12 (30.8%)	12 (23.5%)	9 (56.3%)		

### Laboratory Examinations

**Figure 1:**
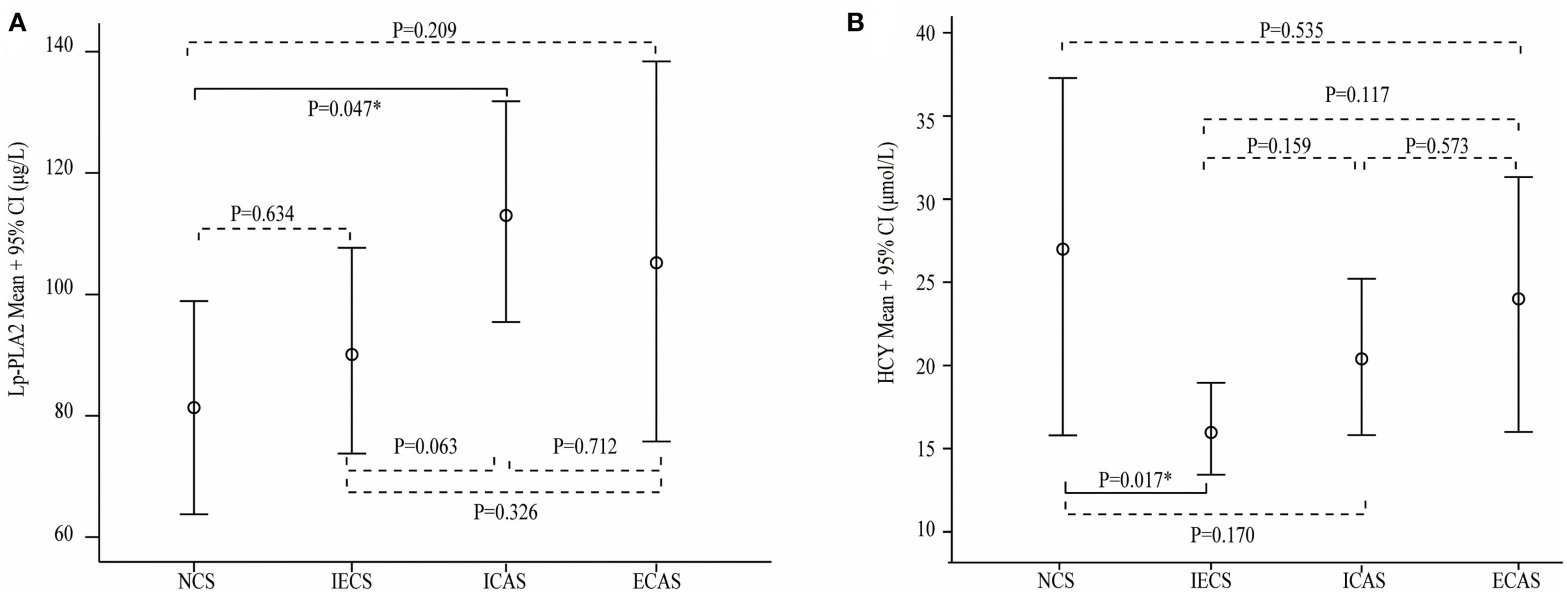
Serum lipoprotein-associated phospholipase A2 (Lp-PLA2) **(A)** and homocysteine (HCY) **(B)** levels in different stenosis subtypes. Lp-PLA2 levels were higher in the intracranial artery stenosis (ICAS) group than that in the other three groups, and the difference between the no cerebral artery stenosis (NCS) and ICAS groups was statistically significant **(A)**. The HCY level was significantly higher in the NCS group than in the intracranial and extracranial artery stenosis (IECS) group **(B)**.

Laboratory parameters between the different stenosis groups are shown in Table 3. Lp-PLA2 levels were significantly different among the NCS, ECAS, ICAS, and IECS groups (81.7 ± 38.5 vs. 106.1 ± 57.8 vs. 112.2 ± 66.8 vs. 89.3 ± 52.2 μg/L, *p* = 0.025). Further statistical analysis indicated that there was a modest but statistically significant difference between the ICAS and NCS groups (*p* = 0.047, Figure 1A). After adjustment for dyslipidemia, coronary heart disease, hyperhomocysteinemia, and TOAST subtypes at baseline, the differences in Lp-PLA2 levels remained statistically significant among the four groups (80.1 ± 15.7 vs. 104.2 ± 15.0 vs. 109.0 ± 9.1 vs. 95.2 ± 10.4 μg/L, *p* = 0.044). Further statistical analyses indicated that Lp-PLA2 levels in the ICAS group were significantly higher than those in the NCS group (*p* = 0.048) after adjustment for the above three factors.

**Table 3:** Laboratory parameters in patients from different stenosis subtypes.

	**NCS (*n =* 20)**	**IECS (*n =* 39)**	**ICAS (*n =* 51)**	**ECAS (*n =* 16)**	**F**	***P***
Lp-PLA2 (ug/l)	81.7 ± 38.5	89.3 ± 52.2	112.2 ± 66.8	106.1 ± 57.8	1.948	0.025
hs-CRP (mg/l)	1.6 ± 0.8	1.9 ± 1.6	2.1 ± 1.6	2.0 ± 1.1	0.406	0.749
ESR (mm/h)	6.8 ± 4.3	10.8 ± 10.2	10.2 ± 10.7	10.4 ± 8.7	0.765	0.561
TC (mmol/l)	4.4 ± 1.1	4.5 ± 0.8	4.4 ± 1.0	4.2 ± 0.8	0.454	0.715
TG (mmol/l)	2.1 ± 1.1	1.6 ± 0.6	1.6 ± 0.7	1.4 ± 0.5	—	0.129
LDL-C (mmol/l)	2.7 ± 1.0	2.8 ± 0.7	2.7 ± 0.8	2.5 ± 0.6	0.633	0.595
HDL-C (mmol/l)	1.1 ± 0.3	1.2 ± 0.2	1.2 ± 0.3	1.2 ± 0.3	1.444	0.053
ApoA1 (g/l)	1.0 ± 0.2	1.1 ± 0.2	1.0 ± 0.2	1.0 ± 0.2	0.741	0.530
ApoB (g/l)	0.9 ± 0.3	0.9 ± 0.2	0.8 ± 0.2	0.8 ± 0.2	0.867	0.460
GLU (mmol/l)	5.6 ± 2.0	6.3 ± 2.6	6.0 ± 2.3	5.1 ± 1.2	1.244	0.297
HCY (umol/l)	27.2 ± 21.9	16.9 ± 10.0	21.5 ± 15.6	24.0 ± 14.8	—	0.018

*TC, total cholesterol; TG, triglyceride; LDL-C, low-density lipoprotein cholesterol; HDL-C, high-density lipoprotein cholesterol; ApoA1, apolipoprotein A1; ApoB, apolipoprotein B; GLU, glucose*.

Similarly, the average HCY level in the IECS group was 16.9 ± 10.0 μmol/L, which was significantly lower than that in the NCS group (27.2 ± 21.9 μmol/L, *p* = 0.017, Figure 1B). However, there was no significant difference when compared with the ICAS or ECAS group (21.5 ± 15.6 and 24.0 ± 14.8 μmol/L, respectively). After adjustment for dyslipidemia, coronary heart disease, and TOAST subtypes at baseline, HCY levels were different among the four groups (26.2 ± 4.0, 23.0 ± 3.9, 21.4 ± 3.9, and 17.9 ± 2.7 μmol/L, respectively, *p* = 0.031). Further statistical analyses indicated that the levels of Lp-PLA2 were different between the NCS and IECS groups (*p* = 0.033) after adjustment.

### Lp-PLA2 Levels and ICAS/ECAS

**Figure 2:**
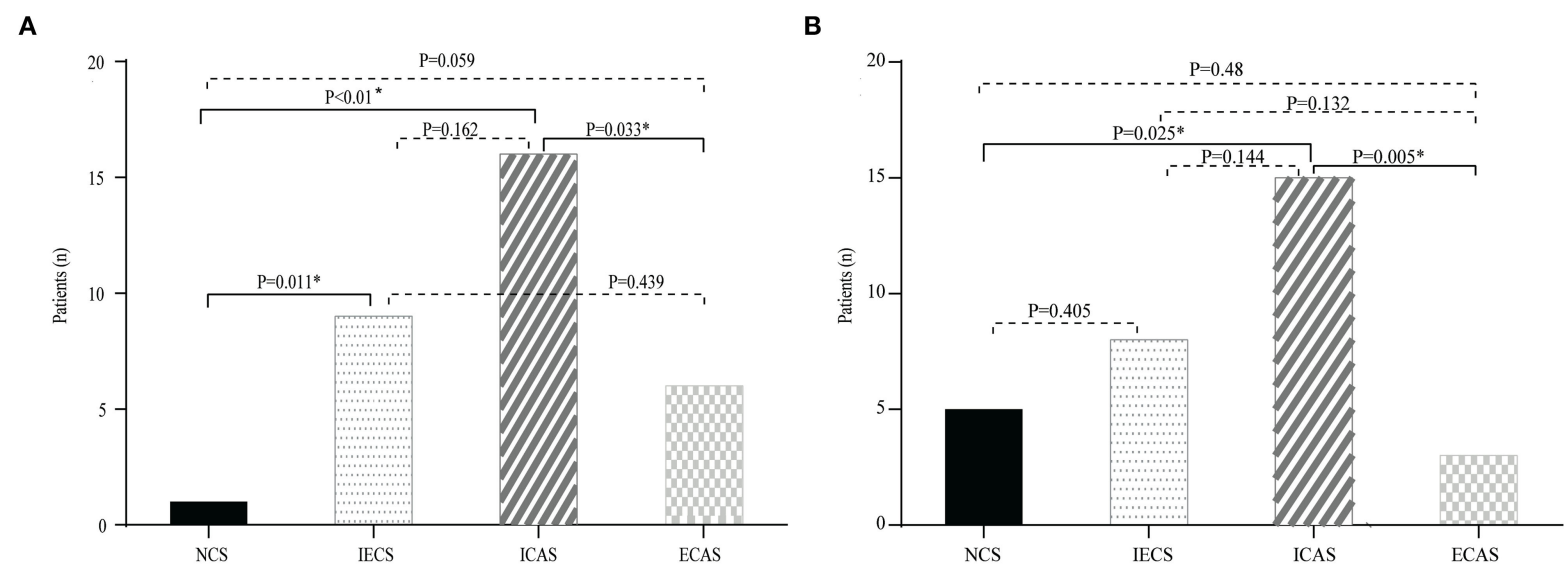
Distribution of different stenosis subtypes in the third **(A)** and fourth **(B)** quartiles of Lp-PLA2. The prevalence of NCS, ICAS, extracranial artery stenosis (ECAS), and IECS according to the level of Lp-PLA2. There was a trend toward an increased risk of ICAS in the third and fourth quartile.

To further investigate whether Lp-PLA2 levels are related to stenosis subtype, we divided Lp-PLA2 levels into four quartiles (Table 4): first (30.8–59.4 μg/L), second (59.4–86.9 μg/L), third (86.9–117.3 μg/L), and fourth (117.3–327.0 μg/L) quartiles. The median Lp-PLA2 mass was 86.9 μg/L (IQR, 59.4–117.3 μg/L). There was no significant difference in the percentage of the four quartiles of Lp-PLA2 levels among the NCS, IECS, ICAS, and ECAS subgroups, although there was a trend toward increased risk of ICAS among participants in the third and fourth quartiles when compared with the first and second [19.6% (first) vs. 19.6% (second) vs. 31.4% (third) vs. 29.4% (fourth), *p* = 0.491]. However, when we performed further statistical analysis of the stenosis subtype in the third and fourth quartiles, we found that, when Lp-PLA2 levels were in the third and fourth quartiles, the proportion of the ICAS group was higher than that of the other three subgroups (third Q: 16[50.0%] vs. 1[3.1%] vs. 9[28.1%] vs. 6[18.8%], *p* = 0.002, Figure 2A; fourth Q: 15[48.4%] vs. 5[16.1%] vs. 8[25.8%] vs. 3[9.7%], *p* = 0.014, Figure 2B).

**Table 4:** Percentage distribution of stenosis subtypes among the four quartiles of lipoprotein-associated phospholipase A2 (Lp-PLA2).

	**NCS (*n =* 20)**	**IECS (*n =* 39)**	**ICAS (*n =* 51)**	**ECAS (*n =* 16)**	**χ2**	***P***
30.8–59.4, *n* (%)	7 (22.6%)	12 (38.7%)	10 (32.3%)	2 (6.5%)	7.323	0.062
59.4–86.9, *n* (%)	7 (21.9%)	10 (31.3%)	10 (31.3%)	5 (15.6%)	2.250	0.522
86.9–117.3, *n* (%)	1 (3.1%)	9 (28.1%)	16 (50.0%)	6 (18.8%)	14.750	0.002
117.3–327.0, *n* (%)	5 (16.1%)	8 (25.8%)	15 (48.4%)	3 (9.7%)	10.677	0.014

### Lp-PLA2 Levels and the Number/Severity of Stenosis Events in ICAS

**Figure 3:**
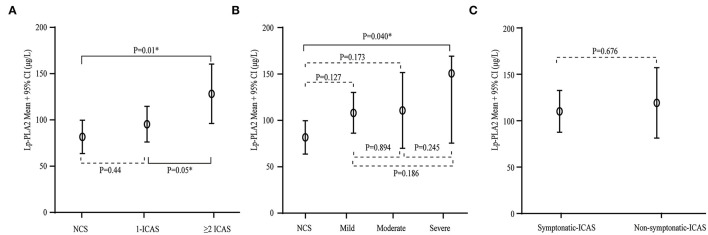
Serum Lp-PLA2 levels and the severity of stenosis in ICAS. **(A)** Serum levels of Lp-PLA2 levels according to the number of ICAS. Compared with those with one lesion and with no arterial stenosis, Lp-PLA2 levels in patients with two or more lesions were higher. **(B)** Serum levels of Lp-PLA2 levels according to the severity of stenosis of ICAS. Lp-PLA2 levels in patients with severe ICAS were relatively higher than in those with moderate or mild ICAS. **(C)** Serum levels of Lp-PLA2 levels according to symptomatic and asymptomatic ICAS. There was no difference in the Lp-PLA2 level between patients with asymptomatic and symptomatic ICAS.

We further investigated whether Lp-PLA2 levels are related to the severity of stenosis, including the number of lesions, the extent of stenosis, and asymptomatic and symptomatic disease in the ICAS group. Patients with two or more lesions had higher Lp-PLA2 levels compared to those with one lesion and with no arterial stenosis (128.3 ± 79.3 vs. 95.5 ± 46.6 vs. 81.7 ± 38.5 μg/L, *p* = 0.025, Figure 3A). Patients with severe ICAS had relatively higher Lp-PLA2 levels than those with moderate or mild ICAS, but there was no statistically significant difference (150.9 ± 100.0 vs. 110.7 ± 70.7 vs. 108.2 ± 61.7 μg/L, *p* = 0.152, Figure 3B). There was no difference in Lp-PLA2 levels between patients with asymptomatic and symptomatic ICAS (110.0 ± 69.4 vs. 119.3 ± 59.8 μg/L, *p* = 0.676, Figure 3C). Patients with higher Lp-PLA2 levels in the ICAS group had an increased risk for multiple or severe stenosis.

## Discussion

In this study, we assessed the association between Lp-PLA2 levels and the location and burden of cerebral atherosclerosis in patients with AIS. We found that patients with ICAS had modestly higher Lp-PLA2 levels than those with NCS and showed a trend for higher Lp-PLA2 levels compared to those with ECAS. Furthermore, a higher Lp-PLA2 level was associated with an increased risk of multiple or severe stenosis in ICAS, suggesting that increased Lp-PLA2 levels might be a risk factor for ICAS.

Lipoprotein-associated phospholipase A2 plays a key role in the pathogenesis of atherosclerosis and has been reported to be positively correlated with subclinical cerebral artery atherosclerotic stenosis, either ICAS or concurrent extra-intracranial stenosis in a stroke-free cohort (8). The present study reveals that serum Lp-PLA2 levels are associated with ICAS in the context of ischemic stroke. The risk of ICAS increased with a higher Lp-PLA2 level in patients with AIS, and patients with higher Lp-PLA2 levels showed more severe ICAS and more intracranial arterial lesions, suggesting that Lp-PLA2 levels are associated with ICAS in both non-stroke and stroke conditions. Therefore, Lp-PLA2 levels should be investigated as a potential biomarker to screen and identify subjects at a higher risk for ICAS in both non-stroke and stroke conditions.

The mechanisms underlying the effect of Lp-PLA2 on atherosclerotic lesions are not entirely understood. One potential mechanism is that Lp-PLA2 participates in the vascular inflammatory process, leading to atherosclerotic lesions. Intracranial arteries are more susceptible to oxidative stress and inflammatory reactions than extracranial arteries due to the absence of an external elastic lamina, the prominent expression of proteasomes that are pro-inflammatory in nature, and reduced expression of inhibitors of inflammation (17). As a proinflammatory and prooxidative molecule, Lp-PLA2 activity and expression levels were associated with the severity of vascular inflammation. Thus, Lp-PLA2 may cause more severe inflammation and oxidative damage in intracranial arteries than in extracranial arteries. Consistent with this, we found that patients with ICAS had higher Lp-PLA2 levels than those with NCS, and a trend for higher Lp-PLA2 levels compared with those with ECAS. Although the specific Lp-PLA2 inhibitor darapladib failed to reduce cardiovascular death, MI, or stroke in two large phase III trials (SOLID-TIMI and STABILITY) in patients with atherosclerosis, Lp-PLA2 might be a rational therapeutic target for ICAS with the development of more potent and selective inhibitors (6).

In our study, patients with AIS in the NCS group were attributed to SVO. The pathogenic mechanisms underlying LAA and small vessel disease remain controversial. Hypercholesterolemia can induce small vessel disease (18). Total cholesterol (TC), which can trigger chronic low-grade inflammation, oxidative stress, and abnormalities in fibrinolysis/coagulation, eventually causes endothelial dysfunction (19) and is linked to a higher frequency and severity of small vessel disease (20). Yu et al. (21) found that the levels of TC and triglycerides were decreased in patients with LAA compared with those in the small vessel disease group. The relationship between lipid profiles with subtypes of vessel atherosclerosis is still under debate. Patient selection, classification of subtypes, and the use of statins may contribute to these differences.

Several previous studies have demonstrated that ICAS and ECAS share similar risk factors, but disparities still exist. Age, hyperlipidemia, and male sex are independent predictors of ECAS, whereas age, unhealthy lifestyles, metabolic disturbance, and hypertension are related to ICAS (22). In line with this, we also found that the ICAS group had a lower frequency of dyslipidemia compared with the IECS group and that the ICAS group was more likely to be symptomatic than the ECAS group. Compared with extracranial arteries, atherosclerosis is more prone to occur in intracranial arteries in Asian and African populations. Among symptomatic patients, the prevalence of ICAD is 33–54% in Asian patients and is around 10% in the Caucasian population (22).

In a meta-analysis of 22 studies involving 157,693 participants, Hu et al. (23) demonstrated that elevated baseline Lp-PLA2 levels, either activity or mass, are associated with increased stroke risk. Lin et al. (24) found that the risk of short-term recurrence increased by 7% for every 30 nmol/min/ml increase of Lp-PLA2 in patients with transient ischemic attack (TIA) in the CHANCE study. Zhou et al. (25) indicated that Lp-PLA2 was independently related to admission severity in patients with AIS, implying a predictive value of Lp-PLA2 for severe cases of stroke. Furthermore, increased Lp-PLA2 levels are not only associated with unfavorable functional outcomes at 3 months and 1 year after AIS (26) but are also an independent predictive factor for all-cause death within 1 year after AIS (27).

As one of the most common causes of stroke, intracranial atherosclerosis is also correlated with a high risk of recurrent stroke. Cao et al. (28) found that the optimal value of Lp-PLA2 levels was 136.46 ng/ml, at which the sensitivity and specificity for the diagnosis of the presence of moderate-to-severe artery stenosis or occlusion were 79.6% and 95.2%, respectively, and the area under the curve (AUC) was 0.938, indicating that the serum Lp-PLA2 level has an excellent diagnostic value for severe stenosis. Wang et al. (29) found that increased serum Lp-PLA2 levels independently predict early neurological deterioration in first-ever AIS patients with large arterial atherosclerosis. In patients with ICAS, Lp-PLA2 elevations were found to be associated with plaque enhancement and surface irregularity, and combined assessments of Lp-PLA2 concentration and plaque enhancement were of greater diagnostic value for the risk of AIS in patients with ICAS (30). Lp-PLA2 levels might be a useful tool to identify patients with ICAS at higher risk of new vascular events (31). For TIA patients with unilateral middle cerebral artery stenoses, Lp-PLA2 levels may also be a promising biomarker to predict plaque vulnerability (32). However, there are few studies on the association of Lp-PLA2 with stenosis subtype in patients with AIS. In our study, we found that higher Lp-PLA2 levels are associated with an increased risk of multiple or severe ICAS and that Lp-PLA2 levels were higher in AIS patients with ICAS than in those with one of the other three groups.

Our study has several limitations. First, the design was cross-sectional, which does not allow the evaluation of the real prognostic impact of Lp-PLA2 levels in patients with ICAS. Second, our data were obtained from a single center in China, and the sample size was relatively small. The incidence of ICAS is higher in Chinese than in other populations (1). Standard errors (SEs) or IQRs vary greatly due to this limited sample size. An increase in sample size and populations may produce more generalizable information, such as whether Lp-PLA2 levels are different between ICAS and IECS groups, and whether Lp-PLA2 is associated with ICAS in other populations. Third, patients with metabolic syndrome have chronic low-grade inflammation and higher Lp-PLA2 activity (33). Increased Lp-PLA2 activity is associated with metabolic syndrome and favors a hypercoagulable and pro-inflammatory state (2). There are several possible mechanisms linking Lp-PLA2 and metabolic syndrome (34), including triggering the production of pro-inflammatory cytokines and inducing and aggravating insulin resistance. All of these increase the risk for both diabetes and atherosclerosis. In addition, Lp-PLA2 could be an indicator of enhanced adipose tissue inflammation, adipokine perturbations, and/or increased fatty acid flux. This may provide a new insight for dissecting the pathogenesis of atherosclerosis in metabolic syndrome and warrants further testing. The fourth limitation of our studies is that blood samples were measured in an acute period of AIS and at variable time intervals from stroke onset, which potentially jeopardizes the validity of our findings. Nevertheless, the Northern Manhattan Study suggested that Lp-PLA2 levels decreased modestly overtime after stroke and remains relatively stable for defining major risk categories (35). Finally, Lp-PLA2 activity was not measured in this study. The Framingham Heart Study suggested that Lp-PLA2 activity and level represent distinct pathophysiological and clinical information (36).

Further studies are needed to elucidate the mechanisms by which Lp-PLA2 promotes increased diffuse intracranial atherosclerosis and to evaluate the impact of Lp-PLA2-lowering therapies on the progression of ICAS. Serum biomarkers alone would be of limited value in predicting stroke risk and prognosis in patients with ICAS. Multimodal imaging technologies can provide a combination of information from the intracranial vessel wall, blood flow in large and intermediate arteries, tissue perfusion, and small vessel disease. These include high-resolution MRI, 3D pseudocontinuous arterial spin labeling, 4D flow MRI, and 7T vessel wall and brain MRI. These technologies may be useful in identifying intracranial atherosclerosis with a higher risk of stenosis or ischemic events (37–39). The combination of blood biomarkers and imaging-based biomarkers, particularly imaging, shows vessel walls at high resolution, will be a future trend in the assessment of the pathogenesis and progression of ICAS as well as potential therapeutic targets, and this warrants further investigation.

## Conclusion

In conclusion, elevated Lp-PLA2 levels were associated with an increased risk of ICAS in patients with AIS, and the level of Lp-PLA2 was higher in patients who had more or severe lesions of ICAS, suggesting that Lp-PLA2 is a promising biomarker for ICAS, and may be a potential therapeutic target for ICAS.

## Data Availability Statement

The raw data supporting the conclusions of this article will be made available by the authors, without undue reservation.

## Ethics Statement

This study was approved by the Ethics Committees of Xuanwu Hospital, Capital Medical University. All participants signed informed consent forms before participation in this study.

## Author Contributions

YW, XJ, and GC: study concept and design. YW, GL, HS, and CC: acquisition, analysis, and interpretation of data. GL: statistical analysis. YW: drafting of the manuscript. YW and GC: critical revision of the manuscript for important intellectual content. All authors read and approved the final manuscript.

## Funding

This work was supported by the Youth Program of the National Natural Science Foundation of China (No. 81801142) and the Beijing Hospitals Authority Youth Program (No. QMS20200801) to YW. GC was supported by Merit Review Grants BX003923 and BX002346 from the Department of Veterans Affairs.

## Conflict of Interest

The authors declare that the research was conducted in the absence of any commercial or financial relationships that could be construed as a potential conflict of interest.

## Publisher's Note

All claims expressed in this article are solely those of the authors and do not necessarily represent those of their affiliated organizations, or those of the publisher, the editors and the reviewers. Any product that may be evaluated in this article, or claim that may be made by its manufacturer, is not guaranteed or endorsed by the publisher.

## References

[1] Wong LKS. Global burden of intracranial atherosclerosis. Int J Stroke. (2006) 1:158–9. 10.1111/j.1747-4949.2006.00045.x 18706036

[2] Wang Y Meng R Liu G Cao C Chen F Jin K. Intracranial atherosclerotic disease. Neurobiol Dis. (2019) 124:118–32. 10.1016/j.nbd.2018.11.008 30439443 PMC6363839

[3] Delgado P Chacon P Penalba A Pelegri D Merino C Ribo M. Temporal profile and prognostic value of Lp-PLA2 mass and activity in the acute stroke setting. Atherosclerosis. (2012) 220:532–6. 10.1016/j.atherosclerosis.2011.11.016 22153151

[4] Donato LJ Meeusen JW Callanan H Saenger AK Jaffe AS. Advantages of the lipoprotein-associated phospholipase A2 activity assay. Clin Biochem. (2016) 49:172–5. 10.1016/j.clinbiochem.2015.09.002 26365697

[5] Meschia JF Bushnell C Boden-Albala B Braun LT Bravata DM Chaturvedi S. Guidelines for the primary prevention of stroke: a statement for healthcare professionals from the American Heart Association/American Stroke Association. Stroke. (2014) 45:3754–832. 10.1161/STR.0000000000000046 25355838 PMC5020564

[6] Huang F Wang K Shen J. Lipoprotein-associated phospholipase A2: the story continues. Med Res Rev. (2019) 40:79–134. 10.1002/med.21597 31140638 PMC6973114

[7] Lp PLASC Thompson A Gao P Orfei L Watson S Di Angelantonio E. Lipoprotein-associated phospholipase A(2) and risk of coronary disease, stroke, and mortality: collaborative analysis of 32 prospective studies. Lancet. (2010) 375:1536–44. 10.1016/S0140-6736(10)60319-4 20435228 PMC2864403

[8] Wang X Wang Y Zhang J Qian Y Tang X Ling H. Association of Lp-PLA2 mass and aysmptomatic intracranial and extracranial arterial stenosis in hypertension patients. Plos ONE. (2015) 10:e0130473. 10.1371/journal.pone.0130473 26098634 PMC4476589

[9] Wang Y Zhou B Zhou P Yao Y Cui Q Liu Y. Association of lipoprotein-associated phospholipase A2 mass with asymptomatic cerebral artery stenosis. J Cell Mol Med. (2018) 22:2329–36. 10.1111/jcmm.13521 29424477 PMC5867129

[10] Sacco RL Kasner SE Broderick JP Caplan LR Connors JJ Culebras A. An updated definition of stroke for the 21st century a statement for healthcare professionals from the American Heart Association/American Stroke Association. Stroke. (2013) 44:2064–89. 10.1161/STR.0b013e318296aeca 23652265 PMC11078537

[11] Chu YW Chen PY Lin SK. Correlation between immune-inflammatory markers and clinical features in patients with acute ischemic stroke. Acta Neurol Taiwan. (2020) 29:103–13. 34018169

[12] Yang X Jia X Ren H Zhang H. The short- and long-term efficacies of endovascular interventions for the treatment of acute ischemic stroke patients. Am J Transl Res. (2021) 13:5436–43. 34150141 PMC8205676

[13] Adams HP Bendixen BH Kappelle LJ Biller J Love BB Gordon DL. Classification of subtype of acute ischemic stroke - definitions for use in a multicenter clinical-trial. Stroke. (1993) 24:35–41. 10.1161/01.STR.24.1.35 7678184

[14] Zhang J Li Y Wang Y Niu W Zhang Y Gao P. Arterial stiffness and asymptomatic intracranial large arterial stenosis and calcification in hypertensive chinese. Am J Hypertens. (2011) 24:304–9. 10.1038/ajh.2010.246 21164493

[15] Sameshima T Futami S Morita Y Yokogami K Miyahara S Sameshima Y. Clinical usefulness of and problems with three-dimensional CT angiography for the evaluation of arteriosclerotic stenosis of the carotid artery: comparison with conventional angiography, MRA, and ultrasound sonography. Surg Neurol. (1999) 51:300–8. 10.1016/S0090-3019(98)00117-7 10086495

[16] Wang J Wu J Zhang S Zhang L Wang C Gao X. Elevated fasting glucose as a potential predictor for asymptomatic cerebral artery stenosis: a cross-sectional study in Chinese adults. Atherosclerosis. (2014) 237:661–5. 10.1016/j.atherosclerosis.2014.10.083 25463102

[17] Qureshi AI Caplan LR. Intracranial atherosclerosis. Lancet. (2014) 383:984–98. 10.1016/S0140-6736(13)61088-0 24007975

[18] Kraft P Schuhmann MK Garz C Jandke S Urlaub D Mencl S. Hypercholesterolemia induced cerebral small vessel disease. PLoS ONE. (2017) 12:e0182822. 10.1371/journal.pone.0182822 28796818 PMC5552130

[19] Shin DW Lee KB Seo JY Kim JS Roh H Ahn MY. Association between hypertriglyceridemia and lacunar infarction in type 2 diabetes mellitus. J Stroke Cerebrovasc Dis. (2015) 24:1873–8. 10.1016/j.jstrokecerebrovasdis.2015.04.030 26004860

[20] Vilar-Bergua A Riba-Llena I Nafría C Bustamante A Llombart V Delgado P. Blood and CSF biomarkers in brain subcortical ischemic vascular disease: involved pathways and clinical applicability. J Cereb Blood Flow Metab. (2016) 36:55–71. 10.1038/jcbfm.2015.68 25899297 PMC4758557

[21] Yu F Lu J Li Z Zhou X Zeng S Zhan Q. Correlation of plasma vascular endothelial growth factor and endostatin levels with symptomatic intra- and extracranial atherosclerotic stenosis in a chinese han population. J Stroke Cerebrovasc Dis. (2017) 26:1061–70. 10.1016/j.jstrokecerebrovasdis.2016.12.021 28189572

[22] Jin H Peng Q Nan D Lv P Liu R Sun W. Prevalence and risk factors of intracranial and extracranial artery stenosis in asymptomatic rural residents of 13 villages in China. BMC Neurol. (2017) 17:136. 10.1186/s12883-017-0924-0 28720076 PMC5516380

[23] Hu G Liu D Tong H Huang W Hu Y Huang Y. Lipoprotein-associated phospholipase a2 activity and mass as independent risk factor of stroke: a meta-analysis. Biomed Res Int. (2019) 2019:8642784. 10.1155/2019/8642784 31236414 PMC6545803

[24] Lin J Zheng H Cucchiara BL Li J Zhao X Liang X. Association of Lp-PLA2-A and early recurrence of vascular events after TIA and minor stroke. Neurology. (2015) 85:1585. 10.1212/WNL.0000000000001938 26311748 PMC4642142

[25] Zhou F Liu Y Shi H Huang Q Zhou J. Relation between lipoprotein-associated phospholipase a2 mass and incident ischemic stroke severity. Neurol Sci. (2018) 39:1591–6. 10.1007/s10072-018-3474-3 29938341

[26] Jiang X Xu J Hao X Xue J Li K Jin A. Elevated lipoprotein(a) and lipoprotein-associated phospholipase a(2) are associated with unfavorable functional outcomes in patients with ischemic stroke. J Neuroinflammation. (2021) 18:307. 10.1186/s12974-021-02359-w 34963487 PMC8715597

[27] Han L Zhong C Bu X Xu T Wang A Peng Y. Prognostic value of lipoprotein-associated phospholipase A2 mass for all-cause mortality and vascular events within one year after acute ischemic stroke. Atherosclerosis. (2017) 266:1–7. 10.1016/j.atherosclerosis.2017.09.013 28934604

[28] Cao J Yan P Zhou Y Zhou X Sun Z Zhu XQ. Clinical utility of the serum level of lipoprotein-related phospholipase a2 in acute ischemic stroke with cerebral artery stenosis. Front Neurol. (2021) 12:642483. 10.3389/fneur.2021.642483 33746893 PMC7969974

[29] Wang Y Hu S Ren L Lei Z Lan T Cai J. Lp-PLA2 as a risk factor of early neurological deterioration in acute ischemic stroke with TOAST type of large arterial atherosclerosis. Neurol Res. (2018) 41:1–8. 10.1080/01616412.2018.1493850 30296199

[30] Yan X Gao J Tang M Zhang D Li L Wang L. Combined assessment of elevated plasma lipoprotein-associated phospholipase a(2) and plaque enhancement improved accuracy in the risk of acute ischemic stroke in patients with intracranial artery stenosis. J Stroke Cerebrovasc Dis. (2021) 30:106103. 10.1016/j.jstrokecerebrovasdis.2021.106103 34587576

[31] Massot A Pelegri D Penalba A Arenillas J Boada C Giralt D. Lipoprotein-associated phospholipase a2 testing usefulness among patients with symptomatic intracranial atherosclerotic disease. Atherosclerosis. (2011) 218:181–7. 10.1016/j.atherosclerosis.2011.04.031 21620406

[32] Qin Y Qian X Luo X Li Y Wang D Jiang J. Association between plasma lipoprotein-associated phospholipase a2 and plaque vulnerability in TIA patients with unilateral middle cerebral artery stenosis. Front Neurol. (2020) 11:574036. 10.3389/fneur.2020.574036 33178116 PMC7596647

[33] Acevedo M Varleta P Kramer V Valentino G Quiroga T Prieto C. Comparison of lipoprotein-associated phospholipase A2 and high sensitive c-reactive protein as determinants of metabolic syndrome in subjects without coronary heart disease: in search of the best predictor. Int J Endocrinol. (2015) 2015:934681. 10.1155/2015/934681 26089902 PMC4452297

[34] De Stefano A Mannucci L Tamburi F Cardillo C Schinzari F Rovella V. Lp-PLA(2), a new biomarker of vascular disorders in metabolic diseases. Int J Immunopathol Pharmacol. (2019) 33:2058738419827154. 10.1177/2058738419827154 30706739 PMC6360470

[35] Elkind MSV Leon V Moon YP Paik MC Sacco RL. High-sensitivity C-reactive protein and lipoprotein-associated phospholipase a 2 stability before and after stroke and myocardial infarction. Stroke. (2009) 40:3233–7. 10.1161/STROKEAHA.109.552802 19644070 PMC2761676

[36] Schnabel R Dupuis J Larson MG Lunetta KL Robins SJ Zhu Y. Clinical and genetic factors associated with lipoprotein-associated phospholipase A2 in the Framingham Heart Study. Atherosclerosis. (2009) 204:601–7. 10.1016/j.atherosclerosis.2008.10.030 19135199 PMC2893025

[37] Liu W Chen Z Ortega D Liu X Huang X Wang L. Arterial elasticity, endothelial function and intracranial vascular health: a multimodal MRI study. J Cereb Blood Flow Metab. (2021) 41:1390–7. 10.1177/0271678X20956950 33081567 PMC8142128

[38] Bjornfot C Garpebring A Qvarlander S Malm J Eklund A Wahlin A. Assessing cerebral arterial pulse wave velocity using 4D flow MRI. J Cereb Blood Flow Metab. (2021) 41:2769–77. 10.1177/0271678X211008744 33853409 PMC8504412

[39] Xu WH Li ML Niu JW Feng F Jin ZY Gao S. Intracranial artery atherosclerosis and lumen dilation in cerebral small-vessel diseases: a high-resolution MRI Study. CNS Neurosci Ther. (2014) 20:364–7. 10.1111/cns.12224 24423003 PMC6493086

